# Modulation of Host Immunity by Helminths: The Expanding Repertoire of Parasite Effector Molecules

**DOI:** 10.1016/j.immuni.2018.10.016

**Published:** 2018-11-20

**Authors:** Rick M. Maizels, Hermelijn H. Smits, Henry J. McSorley

**Affiliations:** 1 Wellcome Centre for Molecular Parasitology, Institute of Infection, Immunity and Inflammation, University of Glasgow, Glasgow, UK; 2 Leiden University Medical Center, Leiden, the Netherlands; 3 Centre for Inflammation Research, University of Edinburgh, Edinburgh, UK

## Abstract

Helminths are extraordinarily successful parasites due to their ability to modulate the host immune response. They have evolved a spectrum of immunomodulatory molecules that are now beginning to be defined, heralding a molecular revolution in parasite immunology. These discoveries have the potential both to transform our understanding of parasite adaptation to the host and to develop possible therapies for immune-mediated disease. In this review we will summarize the current state of the art in parasite immunomodulation and discuss perspectives on future areas for research and discovery.

## Teaser

Parasitic helminths modulate the immune system, preventing immune-mediated ejection and suppressing immune-mediated diseases. In this review, Maizels and colleagues describe the secreted molecules by which parasites achieve this and the methods by which these molecules have evolved.

## Main Text

### Introduction

Helminths are extremely successful parasites, affecting around a quarter of the world’s population (Bethony et al., 2006). They establish chronic infections and if untreated can persist for the lifetime of the host. While the immune system is capable of parasite expulsion (often incurring collateral damage), in natural infections there is frequently a muted immune response in which the host accommodates, and tolerates, the invader. Such re-setting of host immunity results from both host reparative responses to physical damage from tissue-migrating parasites and/or through active immunomodulation by their molecular products. The ability of parasites to defy host immunity reflects their masterful manipulation of the immune system, which as detailed below, is achieved through the release of a spectrum of finely tuned and highly evolved immuno-modulatory factors (Table 1).

**Table 1:** Different Classes of Helminth-Derived Immunomodulatory Molecules

	Abbreviation	Helminth Species	Action	References
**Cytokine & Innate Defense Homologs and Growth Factors**				

Alarmin release inhibitor	HpARI	*Heligmosomoides polygyrus*	Blocks human and mouse IL-33	Osbourn et al., 2017
Asparaginyl-tRNA synthetase	AsnRS	*Brugia malayi*	Structural homology to IL-8, binds IL-8 receptors CXCR1 and CXCR2; chemotactic for neutrophils and eosinophils; induced regulatory responses and IL-10 in a T cell transfer model of colitis	Kron et al., 2013
Chemokine binding protein	SmCKBP	*Schistosoma mansoni*	Neutralizes chemokine activity (CXCL8, CCL3, CX3CL1, CCL2, CCL5); inhibits neutrophil migration but not eosinophil migration	Smith et al., 2005
Granulin-like growth factor-1	OvGRN-1	*Opisthorchis viverrini*	Induces angiogenesis and wound healing (mice); proliferation in human cholangiocytes and pathways associated with wound healing and cancer	Smout et al., 2009, Smout et al., 2015
Helminth defense molecule-1	FhHDM-1	*Fasciola hepatica*	Molecular mimicry of antimicrobial peptide CAP18/LL-37, binds to LPS and reduces its activity; prevents acidification of the endolysosomal compartments and antigen processing; prevents NLRP3 inflammasome activation	Robinson et al., 2011, Robinson et al., 2012
IL-4 inducing principle from *S. mansoni* eggs	IPSE	*S. mansoni; S. haematobium*	Induces IL-4 secretion in basophils via binding to IgE; induces IL-10 in B cells and enhances their capacity to induce Treg cells; translocates to nucleus and reduces bladder hemorrhage *in vivo*	Haeberlein et al., 2017, Kaur et al., 2011, Mbanefo et al., 2018
Macrophage Migration Inhibitory Factor homolog-1	MIF-1	*B. malayi*, *Trichinella spiralis*, *Anisakis simplex*	Induces IL-8 release from monocytes; synergizes with IL-4 to induce alternatively activated macrophages; inhibits experimental arthritis, colitis & allergic airway inflammation via induction of IL-10 & Treg cells	Cho et al., 2015, Park et al., 2009, Tan et al., 2001, Zang et al., 2002
Metalloproteinases	–	*Necator americanus*	Causes proteolysis of eotaxin, but not of IL-8 or eotaxin-2	Culley et al., 2000
Neutrophil inhibitory factor	AcNIF	*Ancylostoma caninum*	Binds β2 integrin CD11b/CD18, inhibit LPS-induced neutrophil migration and release of reactive oxygen species (ROS)	Anbu and Joshi, 2008, Moyle et al., 1994, Zhou et al., 1998
55 kDa glycoprotein	Hcgp55	*Haemonchus contortus*		
T cell immunomodulatory protein	EmTIP	*Echinococcus multilocularis*	Induces release of IFN-γ from CD4^+^ T cells *in vitro*	Nono et al., 2014
TGF-β homolog-2	TGH-2	*B. malayi*	Ligates mammalian TGF-β receptor and suppresses T cell responses	Gomez-Escobar et al., 2000
TGF-β mimic	TGM	*H. polygyrus*	Ligation of TGF-β receptor on T cells leading to induction of Treg cells	Grainger et al., 2010, Johnston et al., 2017
TGF-like molecule	FhTLM	*F. hepatica*	Ligates mammalian TGF-β receptor (albeit with a lower affinity) and induces IL-10 and Arginase in macrophages	Sulaiman et al., 2016

**TLR Signaling**				

ES-62	ES-62	*Acanthocheilonema viteae*	Modulates dendritic cell responses by inducing the selective autophagolysosomal degradation of TLR-transducers (e.g., TLR4) and interaction with MyD88; synthetic variants block inflammation in various disease models, i.e., allergy, rheumatoid arthritis, and colitis	Ball et al., 2018, Goodridge et al., 2007, Pineda et al., 2014, Rzepecka et al., 2014
Fatty acid binding protein	Fh12/15	*F. hepatica*	Suppresses LPS-induced activation via binding and blocking of CD14; induction of alternatively activated macrophages	Martin et al., 2015, Ramos-Benítez et al., 2017
Lysophosphatidylserine	Lyso-PS	*S. mansoni*	Ligation of TLR2 on dendritic cells; licenses DCs to develop IL-10-producing Treg cells	van der Kleij et al., 2002
Mucin-like polypeptide	Fhmuc	*F. hepatica*	Promotes TLR4 activation of DCs and Th1 cell induction	Noya et al., 2016, Noya et al., 2017

**Intracellular Signaling and Gene Expression**				

Abundant Larval Transcript	ALT	*B. malayi*	Upregulates SOCS1, the inhibitor of IFN-γ signaling	Gomez-Escobar et al., 2005
Acetylcholinesterase	AChE	*N. brasiliensis*	Degrades acetylcholine, reduces neural signaling; induces proinflammatory cytokines with diminished type 2 cytokines in transgenic AChE-expressing trypanosome infection	Vaux et al., 2016
*Ancylostoma* secreted protein-2	NaASP-2	*N. americanus*	Binding to CD79A on B cells, downregulation of lyn, PI3K, and BCR signaling	Tribolet et al., 2015
ATP diphosphohydrolase	SmATPDase1	*S. mansoni*	Degradation of the exogenous pro-inflammatory and pro-thrombotic nucleotides ATP and ADP: inhibition of blood coagulation	Da’dara et al., 2014
Calpain-1 & −2	SmCalp-1, SmCalp-2	*S. mansoni*	SmCalp1 and -2 cleave the blood clotting protein fibronectin and high-molecular-weight kininogen (HK)	Wang et al., 2017
Cathepsin L peptidases	FhCL-1,-2,-3	*F. hepatica*	Degrades fibrinogen and fibrin	Mebius et al., 2018
Ectonucleotide pyrophosphatase/phosphodiesterase	SmNPP-5	*S. mansoni*	Ectonucleotide pyrophosphatase/phosphodiesterase homolog, which inhibits platelet aggregation by degradation of ADP	Elzoheiry et al., 2018
α-enolase	OvENO	*Onchocerca volvulus*	Binds to plasminogen and supports plasmin-mediated proteolysis including degradation of host’s extracellular matrix	Jolodar et al., 2003
Inhibitor of potassium channel	AcK1	*A. caninum*	Inhibitor of the voltage-gated channel Kv1.3 in lymphocytes, similar to the polypeptide inhibitor of the sea anemone, Skh; suppressed delayed type hypersensitivity and the proliferation of memory T cells	Chhabra et al., 2014
	BmK1	*B. malayi*		
Omega-1	ω-1	*S. mansoni*	T2 ribonuclease binds to MR and DC-SIGN, after uptake degrades ribosomal and mRNA; primes DCs for enhances Th2 development; enhances IL-1β secretion in macrophages; improves insulin sensitivity	Everts et al., 2009, Everts et al., 2012, Ferguson et al., 2015, Hams et al., 2016, Ke et al., 2017, Steinfelder et al., 2009, Wilbers et al., 2017
	CP1412	*S. japonicum*		
Serine protease-2	SmSP-2	*S. mansoni*	Manipulation host vasodilatation and fibrinolysis: activates tissue plasminogen activator and plasminogen	Leontovyč et al., 2018
16 kDa polypeptide	Sj16	*S. japonicum*	Translocates to the nucleus, induces IL-10 in BM-derived DCs	Sun et al., 2016

**Enzymes and Inhibitors**				

Anti-inflammatory protein-1	AIP-1	*N. americanus*	Family member of tissue inhibitor of metalloprotease (TIMP)-like proteins; suppression in TNBS colitis model: promotes expression of colon IL-10, TGF-β, and TSLP and the accumulation of Treg cells in the colon	Ferreira et al., 2017
Anti-inflammatory protein-2	AIP-2	*N. americanus*	Family member of TIMP-like proteins; suppression in model of allergic airway inflammation via Treg cell induction and suppression of T cell proliferation in cells from house dust mite (HDM)-allergic patients	Navarro et al., 2016
Cystatins	AvCystatin	*A. viteae*	Induction of regulatory IL-10-producing macrophages and hyporesponsive T cells; compromised APC function; reduced airway allergy and intestinal colitic inflammation in mouse models	Daniłowicz-Luebert et al., 2013, Ziegler et al., 2015
	Onchocystatin	*O. volvulus*		Schönemeyer et al., 2001
	LsCystatin	*Litomosoides sigmodontis*		Pfaff et al., 2002
	HpCPI	*H. polygyrus*		Sun et al., 2013
	BmCPI-2	*B. malayi*		Manoury et al., 2001
	Nippocystatin	*N. brasiliensis*		Dainichi et al., 2001
	rSjCystatin	*S. japonicum*		Wang et al., 2016
	rAi-CPI	*Aacaris lumbricoides*		Coronado et al., 2017
	rCsStefin-1	*Clonorchis sinensis*		Jang et al., 2011
Kunitz-type serine protease inhibitor	SjKT-1	*S. japonicum*	Inhibition of trypsin and chymotrypsin, neutrophil elastase, FXa, and plasma kallikrein: both anti-coagulant and anti-inflammatory properties. FhKTM reduces inflammatory cytokine production in DC	Falcón et al., 2014, Ranasinghe et al., 2015
	FhKTM	*F. hepatica*		
Peroxiredoxin	FhPrx	*F. hepatica*	Anti-oxidant enzyme: inactivation of ROS and induction of AA-MF in mouse models	Donnelly et al., 2008
Thioredoxin peroxidase	TPx	*F. hepatica*	Anti-oxidant enzyme, induction of AA-MF with increased IL-10 and PGE2 responses	Donnelly et al., 2005
Tissue inhibitor of metalloprotease	Ace-MTP-2	*A. ceylanicum*	Reduces MHC-I and MHCII molecules on dendritic cells and those DC induce CD4 and CD8 Treg cells	Bąska et al., 2013

**Lipid or Lipid-Binding**				

Protein of *Ascaris suum*-1	PAS-1	*Ascaris suum*	Similar to ABA-1, a nematode tandemly repeated polyprotein with lipid binding properties; inhibitory in an experimental airway allergy model, dependent on IL-10-producing Treg cells and IFN-γ-producing CD8γδTCR T cells	Araújo et al., 2008, de Araújo et al., 2010
Prostaglandin-2	PGE2	*Trichuris suis*	*T. suis* secretes large amounts of PGE2 in a COX-independent pathway; secreted PGE2 is effective in modulating DC responses	Kaisar et al., 2018, Laan et al., 2017, Liu et al., 1992
		*S. mansoni*	Ligation to Dectin1 and Dectin 2 on DC by SEA leads to autocrine production of PGE2 in DC, which lead to increased Th2 polarization	
**Extracellular Vesicles**				

Extracellular vesicles	EV	*H. polygyrus*	EVs contain microRNAs that target epithelial cells and macrophages leading to loss of IL-33 receptor (IL-33R, also termed ST2) expression, and downregulate other immune genes; inhibit eosinophilia in *Alternaria* allergic airway inflammation model	Buck et al., 2014, Coakley et al., 2017
Extracellular vesicles	EV	*S. japonicum*	Induce M1 macrophage differentiation *in vitro*	Wang et al., 2015

**Figure 1:**
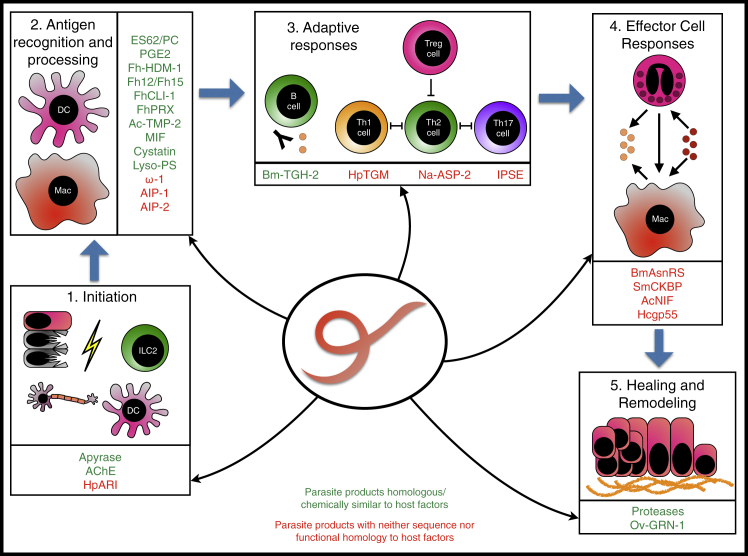
Helminth Modulators Act at All Phases of the Immune Response Modulatory proteins can be homologous to host genes (shared evolutionary ancestry, green) or show no homology (developed *de novo*, red). The immune response is divided into five phases: (1) initiation, innate recognition of damage and danger; (2) innate cell activation and antigen presentation; (3) adaptive immune responses; (4) effector immune responses; and (5) resolution and healing.

Recent advances in helminth genomics and proteomics have uncovered a wealth of such immunomodulatory products. These act on every phase of the immune response, which for the purposes of this review we have divided into: (1) initiation, (2) antigen recognition and processing, (3) adaptive responses, (4) effector cell responses, and (5) coagulation, healing, and remodeling. Parasite immunomodulators active at each phase can be found that either share ancestral homology with host genes or have developed *de novo*, sharing no identifiable homology to the host (Figure 1). Here, we summarize the molecular actors known to interact at each step and their evolutionary and structural provenance, and we discuss future prospects for this exciting field.

### Parasites Neutralize Initiating Alarmin Signals

**Figure 2:**
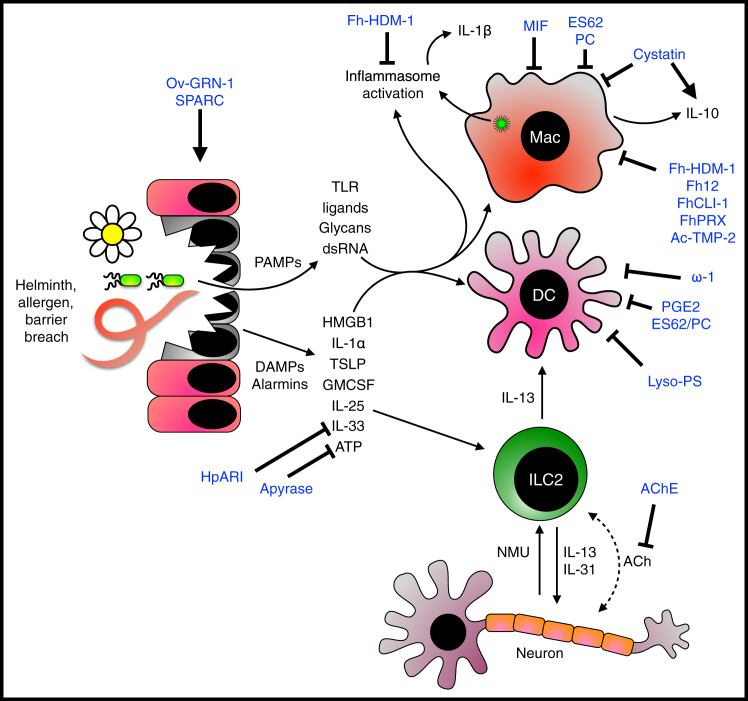
Helminth Modulators that Act on the Early Innate Response to Damage and Danger Helminths and allergens can damage the epithelial layer, resulting in the release of damage associated molecular patterns (DAMPs) and allowing microbial ingress. DAMPs and pathogen associated molecular patterns (PAMPs) can be detected by pattern recognition receptors on dendritic cells and macrophages, resulting in their activation and leading to antigen presentation. Alarmin cytokines such as IL-25, IL-33, and TSLP are also released by epithelial cells and can activate type 2 innate lymphoid cells (ILC2s), which in turn can activate (and be activated by) neurons. ILC2-derived type 2 cytokines aid in the initiation and amplification of the type 2 immune response. Helminth immunomodulators are shown in blue.

Immune responses are initiated by danger signals, through detection of damage-associated molecular patterns (DAMPs) or introduction of pathogen-associated molecular patterns (PAMPs). Parasite migration induces DAMPs and introduces PAMPs from either the parasite itself or bacteria entering tissues through compromised barriers. The imperative to mute barrier surface reactions has driven the evolution of potent mechanisms to suppress innate immune responses (Figure 2).

Activation of epithelial cells results in the release of “alarmin” cytokines such as thymic stromal lymphopoietin (TSLP), interleukin-25 (IL-25), and IL-33. IL-33 is tightly spatiotemporally controlled: it is released on epithelial necrosis and then rapidly oxidized and inactivated (Cohen et al., 2015). The murine intestinal nematode *Heligmosomoides polygyrus* suppresses IL-33 release through HpARI (*H. polygyrus* alarmin release inhibitor) (McSorley et al., 2014, Osbourn et al., 2017) within its excretory-secretory (ES) products. HpARI has a selective mode of action, binding DNA via its first domain, while the second and third domains bind to reduced (active) but not oxidized (inactive) IL-33. Binding obstructs interaction of the complex with the IL-33 receptor, ST2, while interaction with DNA tethers IL-33 within the nucleus of necrotic cells, preventing IL-33 release. HpARI administration ablates type 2 cell-mediated inflammation and improves lung function in an *Alternaria* allergen-dependent asthma model, while in *Nippostrongylus brasiliensis* infection, HpARI administration suppresses type 2 responses and increased worm burden. Crucially, these effects translate to the human setting, as HpARI prevents IL-33 release from human lung explants and blocks human IL-33 release in a transgenic mouse model (Osbourn et al., 2017).

*H. polygyrus* suppresses the IL-33 pathway at multiple levels additionally to HpARI, with an undefined *H. polygyrus* product further downregulating IL-33 production through induction of IL-1β (Zaiss et al., 2013) and through the release of small RNA-containing extracellular vesicles that suppress transcription of the IL-33 receptor (Buck et al., 2014, Coakley et al., 2017).

IL-33 release can be provoked by signals of cell stress or death, in particular extracellular ATP, which induces epithelial and mast cell IL-33 production (Cekic and Linden, 2016). Many parasite secretions contain apyrases (Da’dara et al., 2014, Gounaris et al., 2004), which degrade ATP to non-inflammatory AMP, reducing inflammatory DAMP signals, and hence these could inhibit this important arm of the damage detection response.

### Helminths Target Dendritic Cell and Macrophage Functions

A pivotal point in host immunity is recognition of and reaction to pathogen molecules, typically by pathogen- or damage-associated molecules patterns (PAMPs and DAMPs) ligating pattern recognition receptors (PRRs) on myeloid cells, such as toll-like receptors (TLRs) and C-type lectin receptors (CLRs). These reactions are intensively targeted by helminth molecules (Figure 2), which block TLR ligand-induced dendritic cell and macrophage activation, interfering with receptors and their signaling, as well as antigen presentation and downstream effector mechanisms.

The MyD88 adaptor protein is required for signaling via all TLRs except TLR3, and also IL-1 family cytokine receptors (including the IL-33 receptor). ES-62, a multifunctional glycoprotein secreted by the rodent filarial nematode *Acanthocheilonema viteae* (Pineda et al., 2014), protects against pathology in mouse models of rheumatoid arthritis (RA) (Doonan et al., 2018), asthma (Rzepecka et al., 2014), and lung fibrosis (Suckling et al., 2018). ES-62 induces sequestration of the MyD88 signaling protein, leading to suppression of TLR and IL-33 signaling (Ball et al., 2018, Pineda et al., 2014). The immunomodulatory principle of ES-62 is phosphorylcholine (PC) side groups carried on N-linked glycan moieties (Goodridge et al., 2007), and synthetic small molecule variants of PC can reproduce many anti-inflammatory effects of the parent molecule (Al-Riyami et al., 2013).

A different mechanism is deployed by the immunomodulatory ES protein Fh12 (and its recombinant form, Fh15) from the liver fluke *Fasciola hepatica*. The Fh12 and Fh15 proteins induce alternative activation in human monocyte-derived macrophages (Mo-Macs), suppress macrophage activation by TLR2, TLR4, TLR5, and TLR8 ligands, and inhibit inflammatory cytokine production in septic shock (Ramos-Benítez et al., 2017). They bind to the TLR4 cofactor CD14 and reduce its expression, mediating suppression of TLR4 responses. The Fh12 and Fh15 proteins also suppress responses of CD14-deficient cells, suggesting further inhibitory functions (Martin et al., 2015). Furthermore, cysteine proteases from the flukes *F. hepatica* (FhCL1) and *S. mansoni* (SmCB1) both directly suppress myeloid cell TLR signaling by interfering with TLR-driven inflammation *in vivo*, resulting in the intracellular degradation of TLR3 and TLR4 (Donnelly et al., 2010).

Conversely, some helminth molecules stimulate, rather than inhibit, TLRs. The phospholipid lysophosphatidylserine (lyso-PS) is enriched in the tegument of *S. mansoni* adult worms (Retra et al., 2015). LysoPS is a TLR2 ligand that increases the capacity of human DCs to drive IL-10-producing T cells, suppressing T cell proliferation (van der Kleij et al., 2002). In contrast, the 66-amino acid mucin-like polypeptide from *F. hepatica* (Fhmuc), while not itself a TLR4 ligand, promotes LPS-induced TLR4 activation of DCs, enhancing T helper 1 (Th1) cell responses, both *in vitro* and *in vivo* (Noya et al., 2017).

CLR signaling is important in responses to helminths, and parasite-specific glycans are a rich source of ligands (Hokke and van Diepen, 2017). The schistosome-specific GalNAc-di-GalNAc (LDN) motif is bound by soluble galectin-3 (van den Berg et al., 2004), while fucosylated (LDN-F) derivatives and fucose-containing Lewis X (Le^X^) structures are bound by dendritic cell-specific ICAM3-grabbing non-integrin (DC-SIGN). Mannose-containing structures are recognized by mannose receptor (MR) and macrophage galactose-type lectin (MGL) (van Die et al., 2003). Similar glycan ligands for these receptors are also present in many other parasites including *F. hepatica*. During *F. hepatica* infection, glycan-CLR signaling induces a mixed type 2 and regulatory phenotype with IL-10 and transforming growth factor-β (TGFβ) production (Rodríguez et al., 2017).

Schistosome glycans are strongly immunomodulatory (Meevissen et al., 2012) with a predominant role in Th2 cell polarization through DCs: *S. mansoni* soluble egg antigen (SEA) induces Th2 cell responses *in vivo* in a glycan-dependent manner (Okano et al., 1999). The LNFPIII (lactose-N-fucopentose III) motif, present in both on *S. mansoni* and in human milk, has similar DC-modulating effects as total SEA (Thomas et al., 2005) and can act as an adjuvant for a type 2 immune response (Okano et al., 2001). LNFPIII also stimulates murine B cells to produce the immunoregulatory cytokine IL-10 (Velupillai and Harn, 1994). SEA and human milk also contain LNnT (lacto-N-neotetraose), while intraperitoneally injected milk-derived LNnT induces the influx of a macrophage population that produces IL-10 and TGF-β and suppresses T cell proliferation (Terrazas et al., 2001). Furthermore, schistosome glycans bind to the C-type lectin receptors Dectin-1 and Dectin-2 (Ritter et al., 2010) and prime DCs for enhanced Th2 cell development via autocrine prostaglandin-E2 (PGE2) and OX40L (Kaisar et al., 2018), similar to core α(1-3)-fucose- and β(1-2)-xylose-linked glycans (Faveeuw et al., 2003). Other prominent helminth glycans include chitin, a long-chain polysaccharide that is a potent type 2 response inducer (Van Dyken et al., 2014).

If and when host myeloid cells are triggered through one or more innate receptors, they initiate the proteolytic processing of exogenous antigens and expression of a suite of key stimulatory markers and mediators. Helminths counter with a palette of protein modulators, from protease inhibitors to receptor ligands, that target these pathways. Among the inhibitors, cystatins interfere with cysteine proteases involved in antigen processing, such as lysosomal cathepsins and asparaginyl endopeptidase (AEP). The filarial cystatins, including CPI-2 from *B. malayi*, also specifically inhibit AEP, allowing it to block antigen processing in human cells (Manoury et al., 2001), while LsCystatin (from *Litomosoides sigmodontis*) reduces nitric oxide- and antigen-specific proliferative responses (Pfaff et al., 2002). *In vitro*, Onchocystatin (from *Onchocerca volvulus*) induces IL-10 from human monocytes, together with reduced MHC-II and CD86 expression (Schönemeyer et al., 2001). In mouse models, AvCystatin (from *A. viteae*) similarly drives macrophage IL-10 expression, dampening both airway allergy and colitis (Daniłowicz-Luebert et al., 2013). Furthermore, AvCystatin induces a regulatory PD-L1^+^ and PD-L2^+^ macrophage populations, which on adoptive transfer protect against both airway allergy and intestinal inflammation (Ziegler et al., 2015) and reduce pollen-specific responses in PBMCs from allergic patients (Daniłowicz-Luebert et al., 2013).

Other helminth cystatins with broad immunomodulatory properties including those from *S. japonicum* (Wang et al., 2016), *Ascaris lumbricoides*, and the liver fluke *Clonorchis sinensis* all ameliorate colitis *in vivo* (Coronado et al., 2017, Jang et al., 2011), while Nippocystatin from *N. brasiliensis* blocks responses to ovalbumin (Dainichi et al., 2001). The physiological target of these cystatins *in vivo* has yet to be identified. One possible target might be protease-dependent inflammasome activation, as their effects appear more generalized than interference with the antigen processing machinery alone would predict.

In addition to interfering in myeloid PRR responses, helminths also modulate myeloid cells through mimics of the host’s innate immune messengers. Macrophage migration inhibitory factor (MIF) proteins are evolutionarily ancient, but in mammals they activate myeloid cells. MIF homologs are also found in nematodes (Vermeire et al., 2008): those from *Brugia malayi* (Zang et al., 2002) and *Trichinella spiralis* (Tan et al., 2001) mirror the activity of the host protein, inducing IL-8 release from monocytes, while *B. malayi* MIF also potentiates alternative activation of macrophages, in synergy with IL-4 (Prieto-Lafuente et al., 2009). MIF from *Anisakis simplex* is directly anti-inflammatory, ameliorating both allergy (Park et al., 2009) and colitis (Cho et al., 2015) in mice. Therefore, homology to the host cytokine does not always predict biological activity.

Further macrophage modulation is mediated by a *F. hepatica*-secreted protein termed helminth defense molecule, or FhHDM-1. FhHDM-1 is a homolog of the mammalian cathelicidin-like host defense peptide (HDP) designated CAP18/LL-37. HDPs are antimicrobial peptides that can disrupt bacterial membranes and suppress pro-inflammatory macrophage responses (Tecle et al., 2010). FhHDM-1 binds LPS, preventing LPS ligation and activation of macrophages (Robinson et al., 2011). Furthermore, FhHDM-1 is internalized by macrophages and processed by lysosomal cathepsin L, releasing a short C-terminal peptide which in turn prevents the acidification of the endolysosomal compartment, impairing antigen processing (Robinson et al., 2012) and preventing NLRP3 inflammasome activation and IL-1β secretion (Alvarado et al., 2017). In addition to its immune-modulatory effects, FhHDM-1 may have parasite-intrinsic functions, as it also binds and detoxifies heme (Martínez-Sernández et al., 2017).

More broadly, and beyond signaling pathways and antigen processing, myeloid cell protein expression can be profoundly affected by helminth molecules. For example, ω-1 (Omega-1), a glycoprotein released by *S. mansoni* eggs, is a T2 ribonuclease (RNase). It is taken up by DCs through recognition of its glycan moiety, Lewis-X, by the mannose receptor, and extinguishes protein synthesis through the degradation of ribosomal RNA and mRNA within the cell. Omega-1 does not degrade specific transcripts, but rather targets the global pool of RNA. In this way omega-1, and its close homolog from *Schistosoma japonicum*, CP1412, suppress DC activation and maturation, preventing upregulation of CD86 and MHCII and synthesis of IL-12 in response to CD40 ligation, favoring both Th2 cell (Everts et al., 2012, Everts et al., 2009, Steinfelder et al., 2009, Wilbers et al., 2017) and regulatory T (Treg) cell (Ke et al., 2017, Zaccone et al., 2011) induction. T cell priming in the absence of IL-12 and with diminished co-stimulation may favor Th2 cell polarization.

The Th2 cell-mediated priming capacity of omega-1 has also been confirmed *in vivo* using lentivirus-based transduction to silence gene expression in schistosome eggs, as intravenous injections of those eggs shows reduced lung granuloma formation compared to sham-transduced eggs (Hagen et al., 2014). Lastly, omega-1 induces inflammasome-dependent IL-1β in TLR2-stimulated peritoneal macrophages, which is abrogated when blocking Dectin-1, suggesting that omega-1 can modulate multiple PRR pathways (Ferguson et al., 2015).

The modulatory effects of omega-1 were recently extended to changes in host metabolism. A series of recent studies have demonstrated the beneficial effects of helminth infection on host metabolism (Crowe et al., 2017, Wiria et al., 2014), which can be replicated by helminth products such as SEA (Hussaarts et al., 2015, Wolfs et al., 2014) or recombinant omega-1 (Hams et al., 2016). Injection of omega-1 into obese mice improves insulin sensitivity via IL-33 release from white adipose tissue, which in turn induces ILC2 activation and alternatively activated M2 macrophage differentiation. Mechanistically, the release of IL-33 is mediated by the RNase activity of omega-1, while the improved insulin sensitivity was dependent on the ligation of the mannose receptor (Hams et al., 2016).

Another example showing myeloid cell signaling manipulation is from *B. malayi* abundant larval transcript (ALT). The mosquito-borne *B. malayi* larvae stockpile quantities of ALT proteins prior to entry into the mammalian host. Investigation of gene function by a transfection approach within macrophages identified ALT-induced upregulation of SOCS1, the inhibitor of IFN-γ signaling in inflammation (Gomez-Escobar et al., 2005).

Finally, some helminth proteins contain a nuclear localization signal (NLS) for rapid translocation to the nucleus, including the *S. japonicum*-derived molecule Sj16, which induces IL-10 and inhibits DC maturation following nuclear translocation (Sun et al., 2016). IL-4-inducing principle from schistosome eggs (IPSE) is also rapidly translocated to the nucleus of DCs upon uptake (Kaur et al., 2011), while the IPSE homolog from *S. haematobium* (H-IPSE) translocates to the nucleus of bladder epithelial cells (Pennington et al., 2017). Here, H-IPSE reduces bladder hemorrhage and accelerates urothelial repair in a chemotherapy-induced hemorrhagic cystitis (CHC) model (Mbanefo et al., 2018). The therapeutic effects are IL-4 dependent and may relate to its IL-4-inducing capacity described in basophils (detailed below). The effects of H-IPSE are, however, superior to IL-4 alone, implying that additional nuclear functions are involved (Mbanefo et al., 2018).

The end result of parasite modulation of dendritic cell and macrophage modulation is often aberrant, skewed, or tolerized T cell responses. In the next section we will deal with helminth products that act directly on adaptive immune responses.

### Adaptive Response Modulation Is Targeted at Multiple Levels

**Figure 3:**
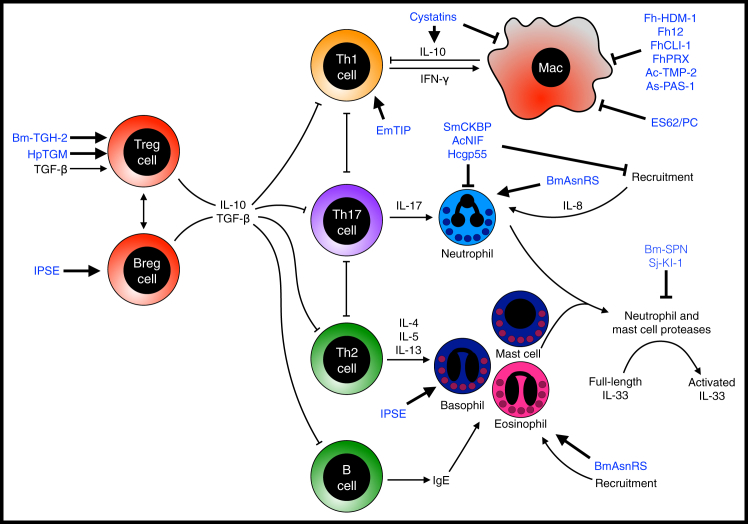
Helminth Modulators that Act on the Adaptive and Effector Immune Responses Immune responses are controlled by helper T cell responses (Th1, Th2, or Th17) and B cell antibody responses. These responses can counter-regulate each other and are suppressed by regulatory T cell or regulatory B cell responses. Effector responses are mediated by T cell-derived cytokines which act on innate effector cells such as macrophages, eosinophils, basophils, and neutrophils. Helminth immunodulators are shown in blue.

On activation by APCs, naive T cells can differentiate into Th1, Th2, Th17, or Treg cell subsets. Modulation of adaptive immune responses has the potential for lasting effects in the host: if the parasite can appear as harmless or commensal, a memory response can be raised that treats the parasite as “self.” Parasitic helminths have developed multiple suppressive and modulatory pathways that suppress effector and/or induce regulatory T helper and B cell responses (Figure 3).

An example of both homologous and *de novo*-generated immunomodulators from helminth parasites can be found in parasite agonists of the TGF-β receptor. The effects of host immune TGF-β signaling are diverse, but are largely immunosuppressive, with generation of induced regulatory T cells, suppression of dendritic cell, macrophage, and T cell activation, and skewing of antibody isotypes to IgA (Chen and Ten Dijke, 2016). Thus, stimulation of the TGF-β pathway would be advantageous to an immunity-shy parasite.

The ancestral function of the TGF-β pathway, across the animal kingdom, is in development: the immunomodulatory role of TGF-β is a more recent adaptive branch within vertebrates (David and Massague, 2018). Notably, ligands and receptors show a high degree of conservation allowing, for example, *S. mansoni* and *Echinococcus multilocularis* TGF-β receptors to ligate human TGF-β family ligands (Beall and Pearce, 2001, Zavala-Góngora et al., 2006). The question is whether TGF-β family members from helminths activate immunosuppressive mechanisms in mammalian cells; indeed, *B. malayi* TGH-2 can signal through the TGF-βR (Gomez-Escobar et al., 2000) while extracts from *L. sigmodontis* also ligate the TGF-βR in a manner that cannot be inhibited with antibodies to TGF-β itself (Hartmann et al., 2015). Finally, a TGF-β homolog from *F. hepatica*, FhTLM, binds host receptors, but with lower affinity and this was inhibited by polyclonal anti-TGF-β antibodies. Immunologically, FhTLM induces IL-10 production in macrophages, indicative of a pro-regulatory outcome (Sulaiman et al., 2016).

In contrast to these instances of evolutionarily conserved members of the TGF-β family activating host receptors, a striking example of convergent evolution has been found in *H. polygyrus.* Secreted products from this intestinal nematode have been shown to activate the TGF-β signaling pathway to induce regulatory T cells *in vitro*, even in the presence of pro-inflammatory cytokines and to a similar degree as TGF-β itself (Grainger et al., 2010). Subsequently, through biochemical fractionation, the factor responsible has been identified as an unrelated structure named TGM (TGF-β Mimic), a 5-domain protein around three times larger than TGF-β and able to bind with high affinity to both TGF-βRI and II, in distinction to mammalian TGF-β which ligates only to RII (Johnston et al., 2017). TGM is also active *in vivo*, for example in prolonging survival of fully allogeneic skin grafts (Johnston et al., 2017). Similarly to HpARI, HpTGM is also effective in cultures of human T cells, despite *H. polygyrus* lacking infectivity in humans.

While the TGF-β pathway directly induces regulatory T cells, other helminth mediators exploit indirect pathways to modulate host T cells. Hookworm-derived homologs of the tissue inhibitor of metalloproteases (TIMPs) modulate DC function, resulting in tolerogenic T cell responses (Cuéllar et al., 2009) and more recently these proteins (now called AIP-1 and AIP-2, for anti-inflammatory proteins) have been demonstrated to be effective quenchers of pathology in mouse models of airway allergy (Navarro et al., 2016) and TNBS-induced colitis (Ferreira et al., 2017). Protection is associated with increased Treg cell and IL-10 responses, alongside abated inflammatory cytokines and modified DC function. Effects are also seen in cultured human DCs and T cells, indicating potential as therapeutics for immune disorders (Navarro et al., 2016).

Treg cell induction *in vivo* is also seen with PAS-1, a 200-kDa native protein purified from *Ascaris suum*, which is reported to inhibit airway allergy in ovalbumin-alum primed mice (Araújo et al., 2008). PAS-1 induces an expands CD25^+^ Treg cell populations, which can suppress allergic responses on adoptive transfer (de Araújo et al., 2010). PAS-1 is similar to ABA-1, a previously described nematode tandemly repeated polyprotein with lipid-binding properties (Antunes et al., 2015, Meenan et al., 2011).

Host galectins promote Treg cell functionality and stability, and a homolog of human galectin-9 from *Toxascaris leonina* reduces DSS-induced colonic pathology in mice with increased production of IL-10 and TGF-β (Kim et al., 2010). While the mechanism is assumed to parallel that of human galectin-9, this has not been verified for the helminth homolog.

Effector T cells are also targeted by certain products. The schistosome glutathione-S-transferase protein (P28GST) appears to promote a sufficiently strong eosinophil-rich Th2 cell response to prevent Th1 and Th17 cell-mediated inflammation in murine colitis in an eosinophil-dependent manner (Driss et al., 2016). In contrast, the *Echinococcus multilocularis* homolog of TIP (EmTIP) induces IFN-γ release from CD4^+^ T cells *in vitro*, which may explain how this parasite, unlike most other helminths, induces an initial Th1 cell response *in vivo* (Nono et al., 2014).

Potassium channels in lymphocytes, such as voltage-gated channel Kv1.3, are crucial for the activation and proliferation of T cells. Kv1.3 is considered an interesting therapeutic target in autoimmune conditions and inhibitors include ShK, a polypeptide toxin expressed in the sea anemone *Stichodactyla helianthus* (Norton et al., 2004). ShK homologs have been conserved and adapted in helminth T cell inhibitors, e.g., AcK1, found in the hookworms *Ancylostoma caninum* and *Ancylostoma ceylanicum*, and BmK1, identified in the filarial worm *B. malayi*. These peptides suppress a delayed-type hypersensitivity (DTH) reaction in rats and inhibit primarily the proliferation of memory T cells without affecting naive or central memory subsets (Chhabra et al., 2014).

An equally important target for helminth parasites is the host B cell response, which is known to be crucial for parasite ejection (Harris and Gause, 2011). Na-ASP-2 (*Ancylostoma* secreted protein 2 expressed by *N. americanus*) binds to CD79A, a component of the B cell receptor (BCR) complex, downregulating mRNA expression of targets involved in signaling and transendothelial migration (Tribolet et al., 2015) while ES-62 from *A. viteae* uncouples the BCR signaling machinery, rendering host cells unresponsive (Deehan et al., 2001). Other types of regulation of B cell responses in helminth infection are associated with a switch from the IgE to the IgG4 isotype, and a regulatory B cell (Breg) phenotype characterized by production of IL-10 (Hussaarts et al., 2011), similar to changes in B cell phenotypes found in allergen tolerance (Akdis and Akdis, 2014).

The secreted products of *S. mansoni* eggs can induce a regulatory B (Breg) cell phenotype in murine and human cells, through or independently of the action of IPSE (Haeberlein et al., 2017). Uptake of IPSE is necessary to induce B cell IL-10 and Treg cell development *in vitro* (Haeberlein et al., 2017). IPSE also supports type 2 cell responses, through induction of IL-4 release from basophils that is IgE dependent but antigen independent and does not require uptake of IPSE by basophils, so this single protein has multiple functions and activation routes (Schramm et al., 2007).

By modulating CD4^+^ T cell differentiation, Treg cell induction, B cell isotype switching, and Breg cell induction, parasites can produce an environment most suited to them. These adaptive responses then determine effector cell responses and whether the outcome of infection is inflammation, resolution, parasite ejection, or tolerance.

### Helminth Molecules Defeat Effector Cell Responses

The full spectrum of cell populations are involved in defense against helminths, from “professional” immune leukocytes to stromal and epithelial cells. In particular, granulocytes (eosinophils and neutrophils) are often the final effector cells in allergic and inflammatory responses, able to target the site of infection and kill pathogens by a variety of means including delivery of toxic compounds, sequestering vital nutrients, and destabilizing the parasite environment. Therefore, parasite survival can be maintained through secretions that block effector cell recruitment and function.

Chemokines are crucial in organizing the immune response through migration of immune cells to sites of inflammation. A key chemokine required for immune cell recruitment and activation in humans is IL-8. *B. malayi* produces asparaginyl-tRNA synthetase (AsnRS) which shows structural (but not sequence) homology to IL-8 and binds the IL-8 receptors CXCR1 and CXCR2, recruiting eosinophils and neutrophils (Kron et al., 2013). However, despite this apparently pro-inflammatory mechanism of action, administration of AsnRS in the mouse T cell transfer model of colitis induces regulatory responses and induction of IL-10 (Kron et al., 2013).

Conversely, human hookworms suppress chemokine responses through production of metalloproteinases which cleave eotaxin-1 (CCL11), but not IL-8 or eotaxin-2 (CCL24), specifically inhibiting the eotaxin-mediated recruitment of eosinophils (Culley et al., 2000). *S. mansoni* secretes a chemokine binding protein (SmCKBP) which binds and blocks IL-8, CCL2, CCL3, CCL5, and CX3CL1—this action inhibits neutrophil (but not eosinophil) migration and has suppressive activity in the DNFB contact hypersensitivity model, while blocking SmCKBP *in vivo* resulting in increased neutrophilia in *S. mansoni* egg-induced granulomas (Smith et al., 2005).

In concert with chemokines, cell migration also requires interaction with integrins such as CD11b/CD18 to facilitate extravasation. One hookworm protein, NIF from *Ancylostoma caninum*, binds this integrin together with fibrinogen and prevents binding of human neutrophils to vascular endothelium (Moyle et al., 1994). NIF is also effective in *in vitro* models of LPS-driven pulmonary inflammation (Zhou et al., 1998), and a NIF-IgE Fc fusion protein (NIF-IGHE-CH4) suppresses human neutrophil migration and blister formation in an *ex vivo* human model of subepidermal autoimmune blistering disease (Kemmer et al., 2015). Remarkably, NIF is a member of a multi-gene family (Pfam00188, variously named CAP, SCP, TAPS, or VAL) that is prominent across a wide range of helminths, and which includes a wide variety of immune response modulators in both animals and plants (Wilbers et al., 2018).

After responding to an infection, the host must heal after epithelial damage and inflammation. This can be mediated by immune pathways and helped (or hindered) by parasite products.

### Helminths Can Promote Healing and Remodeling

As parasites enter the host, they must digest connective tissue to allow their migration, followed by rapid healing to maintain health of their host. They achieve this through secretion of proteases and other “chemical scissors” essential for the invasive process (Ranasinghe and McManus, 2017). Their entry is also facilitated by inhibiting coagulation, particularly prominent in blood-dwelling helminths like schistosomes with multiple strategies to manipulate host vasodilation, fibrinolysis, and coagulation (Mebius et al., 2013). Calpain family proteases SmCalp-1 and SmCalp-2 are expressed in the tegument of *S. mansoni* adult worms, where they appear to cleave and inactivate key coagulation proteins fibronectin and high-molecular-weight kininogen (HK) (Wang et al., 2018).

As discussed above, ATP-degrading enzymes (apyrases) are commonly secreted by helminths, suppressing DAMP-induced inflammatory responses. However, these enzymes have further roles in the control of thrombosis, as extracellular ATP and ADP are potent activators of platelets. Schistosome teguments contain ATP diphosphohydrolase (SmATPDase1) (Da’dara et al., 2014) and the ectonucleotide pyrophosphatase/phosphodiesterase homolog SmNPP5 (Elzoheiry et al., 2018), both of which degrade ATP and ADP to non-thrombotic AMP.

Conversely, a serine protease-2 (SmSP-2) has been found to activate tissue plasminogen activator and plasminogen, both key components of the fibrinolytic system (Leontovyč et al., 2018). Similar activities have been described for other parasite secretions, such as the cathepsin L peptidases FhCL-1, -2, and -3 from *F. hepatica*, which degrade fibrinogen and fibrin (Mebius et al., 2018), and a plasminogen-binding alpha-enolase from *O. volvulus* (OvENO), which may promote proteolysis and degradation of the host extracellular matrix for migration of larvae through host tissues (Jolodar et al., 2003).

Parasite secretions can also encourage the healing of the epithelium. An extreme example of this is the bile duct-dwelling trematode *Opisthorcis viverrini*. This trematode feeds on bile duct epithelial cells and causes extensive inflammation *in situ*. It secretes a granulin homolog (Ov-GRN-1) which causes epithelial cell proliferation: this has potential as a therapeutic agent for non-healing wounds (Smout et al., 2015), but during infection can also result in epithelial transformation and contributes to mortality in *O. viverrini* infection due to cholangiocarcinoma (Smout et al., 2009).

Thus, the secretions of parasitic helminths can modulate every phase of immunity, from the most proximal events in immune initiation to terminal effector responses. In the next section we will discuss how these recent research findings could lead to the discovery of further parasite immunomodulators and how these could be developed for use in human immune-mediated diseases.

### Future Horizons in Helminth Immune Modulation

**Figure 4:**
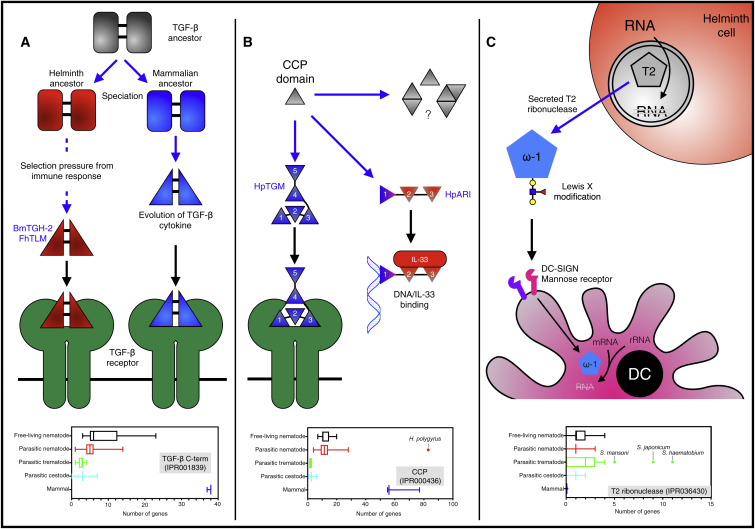
The Evolution and Diversity of Helminth Immunomodulation (A) The TGF-β family is evolutionarily ancient, and nearly all helminth genomes (including free-living helminths) encode TGF-β family members. TGF-β homologs from *Brugia malayi* (Bm-TGH-2) and *Fasciola hepatica* (FhTLM) have co-evolved with their host to bind to the mammalian TGF-β receptor. (B) In *Heligmosomoides polygyrus*, the CCP domain-containing family is over-represented in the genome, and HpTGM (a 5 CCP domain protein) has undergone convergent evolution to bind the TGF-β receptor, despite bearing no homology to the host cytokine. The CCP domain family in *H. polygyrus* appears highly adaptable, and the IL-33-blocking protein HpARI also consists of 3 CCP domains. (C) T2 ribonucleases are involved in a number of homeostatic processes including RNA recycling, and most helminth genomes contain a member of this family. In the schistosomes, however, the T2 ribonuclease family has undergone expansion, and the T2 ribonucelase omega-1 (ω-1) is secreted into the host, where it gains entry to dendritic cells (DCs) through its Lewis X motifs which are bound by glycan receptors. Once inside the DC, omega-1 degrades host messenger and ribosomal RNA, suppressing DC activation and downstream T cell responses. Blue arrows indicate evolutionary processes, black arrows indicate binding. Graphs show number of members of families in each group of genomes (WormBase ParaSite), represented as box and whiskers representing minimum to maximum values.

The field of helminth immunomodulators has, until now, been dominated by protein actors, a tendency likely to continue with the torrent of sequence information extending to more than 100 helminth genomes, each with >10,000 protein-coding genes (International Helminth Genomes Consortium, 2018). Protein sequence analyses also provide insights into the molecular evolution of the different families of helminth immunomodulators (Figure 4). However, this should not overshadow the critical role of non-protein components (alone or in concert with protein moieties), which play many critical roles in helminth immunology.

A relatively neglected aspect of helminth biology has been their production of small molecules with potential for immune modulation, such as prostaglandins and short-chain fatty acids (SCFAs). Some helminths, for example *Ascaris*, release acetate and other metabolites likely to impact responses of the epithelium (Tielens et al., 2010). In addition, helminths can modify the commensal microbiota in a fashion that raises SCFA concentrations, indirectly promoting regulatory mechanisms in the host (Zaiss et al., 2015). With metabolomics now being applied to helminths and genomics piecing together parasite biosynthetic pathways, the suite of such small molecules is likely to now expand rapidly and uncover many more critical interactions in host recognition and immune control. In a recent demonstration of this, small molecules from *A. caninum* suppressed pathology in a mouse model of TNBS-induced colitis (Wangchuk et al., 2018).

Lipid mediators are among the most potent small molecules in the host immune system, both activating and suppressing the immune response. They are <1 kDa, intensely active within their milieu, and have conserved chemical structures across multiple hosts. They represent a target for parasite neutralization or molecular mimicry where helminths would benefit from their effects. PGE2 is an arachadonic acid metabolite, with roles in ILC2, T helper cell, macrophage, dendritic cell, and neutrophil responses (Martínez-Colón and Moore, 2018). PGE2 is strongly linked to Th2 cell and ILC2 responses, for example inducing IL-22-mediated atopic dermatitis (Robb et al., 2018), although it can also directly inhibit ILC2s (Maric et al., 2018). *T. suis*, *S. mansoni*, and filarial nematode secretions contain PGE2 (Laan et al., 2017, Liu et al., 1992), and *S. mansoni* SEA has the ability to induce further PGE2 from host cells (Kaisar et al., 2018), in this case mediating Th2 cell induction.

In the context of the pivotal role of lipid mediators, more attention is likely to be focused on lipid-binding proteins that are numerous among helminth products (Franchini et al., 2015). For example, *F. hepatica* Fh12 (also known as fatty acid binding protein) mentioned above induces alternative activation in human Mo-Macs and suppresses LPS-induced activation via binding and blocking of CD14.

A paradigm-shifting discovery has been that helminths release extracellular vesicles (EVs), often termed exosomes, which are able to fuse and enter host cells, carrying with them a cargo of miRNAs (Buck et al., 2014, Coakley et al., 2017). In *H. polygyrus* infections, these EVs target epithelial cells and macrophages, resulting in their loss of expression of the IL-33 receptor (IL-33R, also termed ST2) and downregulation of other immune genes. These effects result in suppression of both alternative and inflammatory macrophage activation (Coakley et al., 2017). These *in vitro* findings have been corroborated *in vivo*, as *N. brasiliensis* EVs protect against TNBS-induced colitis (Eichenberger et al., 2018), and *H. polygyrus* EVs suppress *Alternaria* allergen-induced allergic airway responses (Buck et al., 2014). In contrast, EVs from *S. japonicum* actively drive M1 macrophage differentiation *in vitro* (Wang et al., 2015), joining a range of helminth-secreted proteins that counter-intuitively induce inflammatory responses (Kron et al., 2013, Noya et al., 2017). Hence EVs are an adaptable part of the armory of helminth immunomodulators, allowing extracellular pathogens to manipulate events within host cells in a receptor-independent manner (Coakley et al., 2017). Further research is required in this area, to investigate the level of specificity of particular EV populations for specific host cells, to determine the relative importance of EV protein and nucleic acid cargoes, and to develop these findings toward potential therapeutic agents for human disease.

### Identification, Characterization, and Clinical Development of the Next Generation of Parasite Immunomodulators

As the identification of defined immunomodulators accelerates, the scale of what the future may hold is also becoming apparent. Each large and complex helminth genome can encode 10,000–50,000 genes (Zarowiecki and Berriman, 2015) (compared to 20,000 in the human genome), and as a result many million previously unannotated genes are now being cataloged. Moreover, parasite secretions are extremely complex, containing hundreds to thousands of protein, carbohydrate, lipid, nucleotide, and vesicular moieties, each of which may be finely tuned to a particular host range.

Parasitism has arisen on multiple occasions within the helminth phyla, and each lineage has evolved specific molecular strategies to defeat the host immune system. As helminths are metazoan members of the animal kingdom, they share many gene families with vertebrates that might provide a template for the molecular evolution of modulators interactive with counterparts in the host. Thus, as the host-adapted genes in the evolution of the immune system, parasites may have followed suit in parallel. Some cases, described below, appear to fulfil this prediction. In contrast, however, some of the most striking parasite immunomodulators such as HpARI, HpTGM, omega-1, and IPSE show no appreciable homology to host immune genes or previously known parasite immunomodulatory factors.

Immunomodulators derived from an ancient shared ancestor of both parasites and their hosts include the TGF-β and MIF families. In the case of TGF-β, a gene family originally required for developmental processes expanded to encompass members with cytokine functions. Thus, as the host developed an immunological role for TGF-β, some parasites have been able to adapt pre-existing developmental homologs toward an immune-modulatory role. Therefore, the TGF-β homologs encoded in parasite genomes do not necessarily indicate immune modulatory functions, while conversely a single TGF-β homolog may fulfil both developmental and immunomodulatory roles (Figure 4A).

It was recently discovered that, rather than adapt a pre-existing TGF-β homolog to interact with host receptors, *H. polygyrus* has convergently evolved an unrelated structure in the form of Hp-TGM, a protein consisting of five atypical complement control protein (CCP) domains with no homology to TGF-β, which ligates host TGF-β receptor (Johnston et al., 2017, Smyth et al., 2018). Furthermore, the same parasite blocks IL-33 through HpARI, another CCP domain protein (Osbourn et al., 2017). Although CCP domains are present in all helminths studied, there has been a preferential expansion of these genes in *H. polygyrus* (Figure 4B). CCP domains occur in a wide range of organisms and serve diverse functions in neuronal signaling, cytokine receptors, hemoglobin binding, and of course, complement control. CCP domain proteins are highly stable and soluble (each tight, globular CCP domain contains two disulfide bonds and conserved glycosylation), structurally simple but functionally sophisticated (Makou et al., 2015) with independent activities of multiple domains within a single protein. This adaptable scaffold appears to have been exploited by *H. polygyrus* in particular.

Gene family expansion in helminths hints at functional adaptation to the parasitic life style. Among the most notable is Pfam00188, which in helminths include venom allergen-like (VAL) and *Ancylostoma* secreted (ASP) proteins. This gene family is dramatically expanded in many parasitic nematodes (especially the hookworms and strongylides) and have been implicated in lipid binding, immune modulation, and pathogen resistance, so they may, like the CCP domain family, represent an adaptable scaffold upon which a range of functions can be developed (Wilbers et al., 2018).

Conversely, expansion of the T2 ribonuclease family (containing omega-1) (Everts et al., 2012) appears specific to schistosomes (Figure 4C), implying that this functionality may have evolved within the trematode family relatively recently. T2 ribonucleases are broadly expressed across many parasites, plants, and animals and are implicated in recycling of RNA and in RNA degradation during the stress response. T2 ribonucleases typically enter the secretory pathway and are often found in vacuoles and autophagosomes within cells. However, some T2 ribonucleases (for instance E^rns^ from pestiviruses) can also be secreted (Luhtala and Parker, 2010), for degradation of RNA in extracellular spaces or in target cells, and it is this latter function which appears to have been utilized in the schistosomes, degrading RNA in host DCs and suppressing their maturation (Everts et al., 2012). Thus, schistosomes have “weaponized” a normal metabolic pathway to suppress DC maturation.

Similarly the apyrase family, which is expanded in many parasitic nematodes (International Helminth Genomes Consortium, 2018), may have originally been a metabolic enzyme but have subsequently used to suppress of host damage detection and inhibit blood coagulation (Da’dara et al., 2014, Gounaris et al., 2004).

These examples of helminth modulation exemplify the techniques used by helminths to develop immunomodulatory products: convergent evolution of shared protein families, *de novo* production of cytokine mimics or antagonists from adaptable modular domains, and hi-jacking of pre-existing protein functions. The agenda for the future is to test whether administration of defined helminth-derived molecular entities (with known functions and in a specific context) can lead us to clinically effective therapeutic molecules.

### Molecular Therapies from Helminths—Where Are We?

In 2005, the first helminth therapy trials (using live *T. suis* infection) showed impressive effects in IBD (Weinstock and Elliott, 2013) and generated a great deal of interest in the use of live helminths as therapeutic modalities. However, subsequent trials have shown smaller or absent effect sizes (Feary et al., 2010, Fleming et al., 2017, Schölmerich et al., 2017), and a lack of understanding of the mechanistic underpinning of helminth immunomodulation has hampered progression. With the recent advances in understanding of helminth immunomodulation at the molecular level, enormous potential exists for helminth-derived products for clinical use.

ES-62 was one of the original immunomodulatory molecules identified from parasites and with its activity through the small molecule phosphorylcholine (PC) moiety, it has great potential for therapeutic drug-like molecules. Two small molecule analogs (SMAs) of PC, termed 11a and 12b, can replicate many of the suppressive effects of ES-62, and due to their small size, stability, and lack of immunogenicity and efficacy are now being developed as therapeutic agents (Al-Riyami et al., 2013). Similarly, many of the recently identified immunomodulatory proteins are likely to be pursued as potential anti-inflammatory therapeutics for clinical use. These proteins have evolved to function in the mammalian milieu and represent natural biologicals that may offer unique mechanisms of action not available with existing drugs; in this context, the coming years of translational research promise to be very engaging.

We summarize here a remarkable panoply of helminth molecules which, when tested in isolation *in vitro* or *in vivo*, target distinct host populations and pathways. Little is yet known, however, of how products from parasites in specific niches (such as the intestinal lumen) are able engage the broader host immune system, whether they exploit uptake mechanisms for systemic dissemination or rely on localized effects in their preferred site. Certainly, in species such as *H. polygyrus,* secretion products differ markedly between immature tissue-dwelling larvae and the intestinal lumen-dwelling adults (Hewitson et al., 2013), arguing for developmental adaptability for interaction with the immune system. In other cases, blood-dwelling parasites such as schistosomes or lymphatic-dwelling filarial worms have more direct access to immune cells and may more readily exert a systemic effect.

Although parasite immunomodulators may act potently in laboratory tests, one obstacle to clinical development has been potential immunogenicity as foreign proteins *in vivo*. However, immunogenicity of helminth immunomodulators would represent an adverse outcome for the parasite, and thus mechanisms may be in place (including the innately immunosuppressive nature of these molecules) for avoidance of this: the fully foreign hookworm product AIP-2 is minimally immunogenic *in vivo* (Navarro et al., 2016).

There are also good precedents for the development of pathogen-derived proteins that have been successfully developed for human use, despite concerns of immunogenicity. Streptokinase (a *Streptococcus*-derived thrombolytic) has been used for decades in thrombotic disease such as myocardial infarction. Although highly immunogenic, as long as the drug is not repeatedly administered, it is effective and its immunogenicity does not present a barrier to use (Sikri and Bardia, 2007). Likewise, the myxoma viral protein Serp1 is an anti-inflammatory inhibitor of serine proteases; administration of Serp1 causes a non-neutralizing antibody response and subsequently the functional activity of the protein has been extended (and potential immunogenicity decreased) through development of small peptide mimetics that replicate the function of the full protein (Ambadapadi et al., 2016). Therefore, formulations of helminth immunomodulators are being developed in which immunogenicity is not present, or is not a barrier to use, for future therapeutic applications.

### Conclusions

Helminth immunomodulation is coming of age. Through advances in -omics analysis of parasitic helminths, ever more information about helminth products is becoming available. However, only by careful analysis of defined recombinant products can the potential of helminth therapeutic agents be realized. In recent years this has led to the characterization of a handful of helminth immunomodulators that could be developed for use in the clinic. Considering the size and complexity of helminth secretions, the complex interaction with their host, and the intricacy of the immune response directed against them, it appears almost certain that we have only scratched the surface of the full gamut of helminth immunomodulators, and that many more will be identified in the next decade.
